# The Diseases of the Male Urethra

**Published:** 1897-04

**Authors:** 


### Book Reviews.

THE DISEASES OF THE MALE URETHRA. By R. W. STEWART, M. D., M. R. C. S., Surgeon to Mercy Hospital, Pittsburg, Pa. One volume of 229 pages, post-octavo, illustrated by numerous wood-engravings. New York: William Wood & Co. 1896.

The neglect of venereal and genito-urinary diseases by the regular profession for many years left an open field for the exploits of the quack, the charlatan and the pretender. A marked change, however, has taken place, so that today these maladies receive special and exclusive attention of eminent and learned medical men. This is indicated by the large volume of transient literature and numerous publications concerning these subjects. One of the latest is the treatise now under consideration. It has been stated that this is the only medical book ever written by a Pittsburg doctor. If this is true, the profession of the Smoky City need not be ashamed of its first-born.

Dr. Stewart, in the introduction, says that the invention of new instruments and the advancement of our knowledge ushered in a new era in urethral pathology and improved methods of treatment. He discusses the acute and chronic inflammation and stricture of the urethra, in which he also includes the inflammatory diseases of the principal glands communicating with that outlet.

Chapter I. is a clear and concise presentation of the anatomy of the urethra. Fig. 3 is an original schematic diagram illustrating how distension of the bladder opens up the internal vesical sphincter. More or less distension of the bladder, according to its condition, causes rhythmic action of the vesical muscles, resulting in dilatation of the internal sphincter (see Ott's experiments with the bladder of a cat), thus permitting urinary invasion of the vesical neck (often miscalled the prostatic urethra), which arouses the desire for urination.

Chapter II. treats of gonorrhea, in which the author recognises this malady as of very ancient pedigree and as prevalent as vice. The accumulative evidence that the gonococcus of Neiser is the cause of gonorrhea is so convincing that the subject has passed the debatable stage.

The causes of acute urethritis are gonococci, other microörganisms, mechanical and chemical excitants. Gonorrhea is no respecter of persons. It attacks alike the prince and the pauper, the reverend and the roué. This malady is acquired by sexual contact with a gonorrheic female, yet she may present no signs recognisable by inspection. The female may remain immune and still transmit the disease from a gonorrheic adorer to his follower. The period of incubation is from two to twenty days. Treatment of this malady has not kept pace with its pathology and remains where our fathers left it. In spite of the discovery of its cause, the numberless remedies proposed and the numerous sure and prompt cures exploited by enthusiasts, numerous urethræ continue to weep. We have not yet found the Balm of Gilead for this disease. The pathology as given by Finger is quoted and elaborated.

Dr. Stewart, in the light of pathology, denies the causative relationship of stricture and gleet, both results of granular urethritis, gleet being one of its earliest symptoms, while stricture does not manifest itself for months, and often years, after the inception of the gonorrhea. Guion reports 142 cases of stricture, forty-nine appearing from ten to fifteen years after the gonorrhea. In the experience of the reviewer, gleet may continue for years, characterised by more or less profuse discharges and decided weakness of genital function, without the formation of a stricture of any degree. Granular tissue causes gleet, and its conversion into stricture tissue cures gleet. The only relationship betwixt gleet and stricture is their common origin. One of the characteristics of chronic specific urethritis is its proneness to exacerbations incited by venery, intemperance, over-eating and over-exertion. Venery causes a profuse discharge, which will subside in a few days, with or without treatment. These are the cases cured in a few days by favorite prescriptions and boasting doctors.

The laws formulated by Otis, that the caliber of the urethra bears a definite relationship to the circumference of the penis, and that stricture is the progenitor of gleet, have done great injury to urethral surgery. Otis claimed that stricture maintained gleet. He did not accuse it of begetting that pervert. "The circumference of the penis is a very variable quantity, even in the same urethra." (?)

Stewart has devised a new urethrometer—an ingenious and practical instrument. The author sums up the treatment of chronic anterior urethritis as follows :

Local treatment should not be attempted during an exacerbation of the inflammation. The urethra should be restored to its normal caliber and resiliancy by gradual dilation with sounds. The meatus should be cut, if necessary, but internal urethrotomy should be avoided when possible, on account of its mortality. If the urethra is not too sensitive, local treatment with the endoscope should be employed in conjunction with gradual dilation.

The use of injections is to be governed by the degree of catarrhal inflammation. Gonorrheics may marry when the gonococci are not found in the urethral secretion, after repeated examinations. This will occur, according to the researches of Goll, in from five weeks to three years.

Chapter XXI. gives a clear, terse description of the anatomy of the posterior urethra, so compact that it cannot be epitomised.

Acute posterior urethritis is usually a sequence of gonorrhea, and, according to the observation of Heissler in fifty cases, it appeared as follows : First week after infection, 20 per cent.; second, 34 per cent.; third, 14 per cent.; fourth, 29 per cent.

Finger says that it does not come on so early if the acute anterior urethritis is not treated locally. Cowper's glands are analogous to the glands of Bartholin, and their function is sexual. Acute inflammation of Cowper's glands is generally the result of a gonorrhea, and is frequently overlooked or its symptoms attributed to some other cause. Abscesses within these glands should be punctured. Some endoscopists claim to be able to make applications to the excretory duct of these glands.

Chapter XXII. relates to stricture of the urethra, and contains several original cuts, to illustrate urethral anatomy. The author states the following conclusion :

The caliber of the urethra depends on the degree of urethral distension, being approximately uniform if the distending force is slight; but showing marked irregularities on forced distension, so that no one part can be taken as a criterion of the dimensions of another.

The circumference is variable, and bears no fixed ratio to the urethral lumen.

Treatment of stricture is conservatively and clearly outlined, and preference is given to dilatation instead of urethrotomy. This volume is nicely printed and bound ; its contents are prepared by one who knows whereof he speaks, who is independent in thought, original in design, and who makes his ideas plain without being prolix. B. H. D.

